# Epigenetic biomarkers of mortality risk in mice under chronic social stress

**DOI:** 10.1007/s11357-025-01721-7

**Published:** 2025-06-09

**Authors:** Samuel D. Anderson, Maria Razzoli, Brian Chen, Matteo Pellegrini, Alessandro Bartolomucci

**Affiliations:** 1 Leonard Davis School of Gerontology, University of Southern California, Los Angeles, CA USA; 2 Department of Integrative Biology and Physiology, University of Minnesota, Minneapolis, MN USA; 3 California Pacific Medical Center Research Institute, Sutter Health, San Francisco, CA 94143 USA; 4 Department of Molecular, Cell and Developmental Biology, University of California, Los Angeles, USA

**Keywords:** DNAm, Chronic Social Stress, Blood Glucose, Accelerated Aging

## Abstract

A strong association exists between exposure to life stressors and accelerated aging in humans and animal models. However, the molecular mechanisms that underlie the adverse effect of stress on aging remain poorly characterized, and there is a paucity of prognostic predictors of stress-induced disease outcomes and life expectancy. To address this gap, we developed mathematical models to predict remaining lifespan based on healthspan data across two independent cohorts which were part of a large study (350 + mice) on social stress and aging in mice. We then relate remaining lifespan to changes in DNA methylation, due to its strong association with age as well as environmental factors such as stress exposure. Multivariate multiple regression identified blood glucose as a major trait associated with DNA methylation. An independent neural network analysis also identified blood glucose among the traits most associated with mortality risk. Finally, elastic net regression identified several DNA methylation sites, including Ptp4a3, Lrrc3b, Adgrb1, Mron5, and Gm6549, which represent possible targets at the intersection of glucose, stress and survival. Overall, the main finding of our analysis is that epigenetic biomarkers of mortality risk reveal an association with blood glucose levels, informing on individual life trajectories shaped by the impact of chronic social stress.

**Supplementary Information:**

The online version contains supplementary material available at 10.1007/s11357-025-01721-7.

## Introduction

Compelling evidence from epidemiological studies describes the relationship between lifelong social disparities, low socio-economic status (SES), and debilitating non-communicable chronic diseases [1–6]. Social studies also show an association between chronic social stress and age acceleration/lifespan shortening [7–9]. However, both connections remain so far without a clear biological explanation. Some important advancements to infer causation have been made [10, 11], yet a more precise understanding of the causal underpinnings of the relative risk conferred by low SES and life stress remains unclear in the absence of randomized experiments, which are impossible and unethical in humans [3, 12].

Animal models are often used to recapitulate several aspects of human biology, including the reproducibility of the association between the animal equivalent of low SES and decreased survival [6, 13–18]. However, only rarely do datasets from animal models of social determinants of health have the depth and complexity necessary to approximate the quality of clinical studies [6, 19–22]. Additionally, studies linking epigenetic changes to mortality risk in animal models relevant to social determinants of health and aging are limited [6, 23–25]. Prior research identifying age and life expectancy algorithms has primarily aimed at associating lifespan and disease states with changes in healthspan and epigenetics generally, or in the context of pre-defined cellular pathways or frailty-related indexes [21, 26–29], with comparatively little research investigating the dynamic interplay between these factors—for instance, how specific, potentially transient shifts in particular healthspan traits at certain time points may align with epigenetic modifications tied to mortality risk.

In the last decades, technological and computational breakthroughs have revolutionized the extent to which biological samples can be interrogated taking greater advantage of ever-growing information dense data. In particular, the development of high throughput genomic technologies has enabled the measurement of genetics, epigenetics and transcriptomics at unprecedented levels of detail [30, 31]. These datasets can then be analysed with sophisticated analysis approaches such as neural networks, a tool which, through mimicking the process of human learning, can identify non-linear relationships between parameters. Here we took advantage of an existing dataset [6, 18] to model the relationship between survival, healthspan, and DNA methylation (DNAm) in mice exposed to chronic psychosocial stress. Deep phenotyping included aggressive behavior, metabolic assessment during the lifecourse from 300 + animals belonging to 3 laboratory strains of mice, liver DNAm extracted from a subset of those mice sacrificed after 17 months, and finally organ abnormalities at necropsy and age at death [6, 18]. We previously reported a negative association between lifelong psychosocial stress and healthspan and lifespan trajectories using this same dataset [6, 18], yet the link between those aging-relevant variables remains unknown.

To help bridge this gap, we trained a multilayer perceptron [32] to predict remaining lifespan based on healthspan for the subset of mice possessing these datatypes, then applied this model to a separate cohort of mice possessing healthspan and DNAm acquired post-sacrifice, generating remaining lifespan predictions which we then regressed against the DNAm. This novel approach allowed us to inform the relationship between chronic social stress, healthspan, and mortality risk in a way unattainable using either group in isolation, due to their forked protocols, and contextualize this epigenetically. We then combined this approach with a separate model intended to identify those CpG sites and traits most strongly predictive of one another across various timepoints. Our approach provides a unique inference on what could be underlying traits, genes, and transcription factors linked to diverging life trajectories due to the interplay of psychosocial stress and genetic background.

## Methods

### Animals and study design

Data included in the present study were previously published in [6, 18]. As previously described [18], C57BL/6J (Jackson Labs), CD1 (Charles River Labs), and Sv129Ev (Taconic Farms) male mice were purchased at 10 weeks of age to be allocated to each experimental condition at 12 weeks of age. All animal procedures were approved by the IACUC, University of Minnesota. Briefly, the experimental conditions were based on the lifelong chronic psychosocial stress (LCPS) model that consisted of a baseline phase of 5 days, during which all mice were singly housed, followed by a 4-week CPS phase in which mice were exposed to daily defeats and sensory contact housing, and an aging phase lasting until spontaneous death or euthanasia for humane reasons. In the aging phase, mice were housed in sensory contact thus experiencing a continued degree of threat stress related to the previous encounters. Each C57BL/6J (N = 173) male mouse was randomly transferred as an intruder to the home cage of either a CD1 (N = 86) or Sv129Ev (N = 87) resident mouse, exploiting the aggression gradient of the two strains [33–35].

The following measures were taken: aggression exhibited and received during the daily social interactions were measured and summarized using a previously described aggression index as well as utilizing the conventional social rank assignment of subordinate or dominant, with individuals of uncertain classification designated as unknown [6]; body weight (in g) and food intake (in kcal), body composition (fat mass and fat free mass, both in g, Echo MRI 3-in-1, Echo Medical System), and 4 h fasting plasma glucose (in mg/dL, Accu-chek Aviva, Roche) were recorded regularly; mortality as the age at death (months) was recorded to the nearest date. All data can be accessed at Mendeley Data (10.17632/kfkhgw359t.1).

### DNA methylation sample preparation and array processing

In a second experiment 70 C57BL/6J, 35 CD1 and 35 Sv129Ev mice purchased from the respective vendor at 10 weeks of age were exposed to LCPS. Mice were sacrificed at 17 months of age for the collection of tissue specimens, which were processed following manufactured instructions. All methods are described in [6]. The DNAm data is deposited in the NCBI Gene Expression Omnibus (https://www.ncbi.nlm.nih.gov/geo/query/acc.cgi?acc=GSE216631).

The DNA methylation assay was performed using the Infinium Mouse Methylation Beadchip at the University of Minnesota Genomics Center (https://genomics.umn.edu/) according to the standard manufacturer’s protocol (http://illumina.com/). Raw intensity data files (.idat files) were processed using GenomeStudio (2011.1) as described in [6].

### Data Analysis Construction

Downstream data analysis was performed in Python, with code available at https://github.com/SamAndTheSun/CSS_healthspan_epigenetics. Two distinct models were developed. The first utilized multiple multivariate regression (MMR) followed by multiple linear regression (MLR). The second utilized a neural network (multi-layer perceptron; MLP) followed by elastic net regression. In both models the “strain” of each respective animal was encoded across two true/false traits to avoid a linear relationship between strains: “C57BL/6J or Sv129Ev” and “CD1 or C57BL/6J”. “Rank” was encoded categorically such that 0 = submissive, 1 = neither dominant nor submissive, and 2 = dominant.

### Model 1: Multiple Multivariate Regression and Multiple Linear Regression

The observed methylation status at site *s* for an animal *a* can be modelled as a combination of *t* methylation associated traits weighted by site specific coefficients:

$${M}_{a,s}^{o}={T}_{a,t}{C}_{t,s}$$

Here, ${M}_{a,s}^{o}$ represents the measured methylation level of site *s* in animal* a*, ${T}_{a,t}$ represents an individual trait associated with the methylation status at site* s*, and ${C}_{t,s}$ represents the site-specific coefficient. The site coefficients ${C}_{t,s}$ are characteristic of the site, shared amongst all animals, and can be determined as ${C}_{t,s}= {{T}_{a,t}}^{+}{ M}_{a,t}^{o}$, where ${{T}_{a,t}}^{+}$ is the Moore–Penrose pseudoinverse of ${T}_{a,t}$. Once the site-specific coefficients are obtained, we can also predict the values of the traits of interest as ${T}_{a,t}^{pred}{={{M}_{a,t}^{o} C}_{t,s}}^{+}$, and the predicted value of the methylation matrix,${M}_{a,s}^{pred}={T}_{a,t}{C}_{t,s}.$

The trait values were first standardized by dividing each trait by its standard deviation across all animals. We then, to minimize collinearity amongst traits, sorted the traits by time point (descending) and constructed a symmetric matrix with dimensions 55 × 55, with row *i* and column *j* each representing the same trait for *i* = *j*, with the values of the matrix being the Spearman correlation between any two intersecting traits. We then masked the values on and above the diagonal, with the exception of the rank and strain columns. We then additionally masked the rank and strain rows, due to their importance in this model, and removed all rows which had any value greater than 0.7. This resulted in 36 traits being removed and 19 being maintained.

Next, to identify those sites that are both variable among individuals and well predicted by our model, the methylation matrix ${M}_{a,s}^{o}$ (46 animals × 284,860 sites) and the trait matrix ${T}_{a,t}$ (46 animals × 19 traits) were utilized in the above formulas. After appending an additional column of ones to the trait matrix to account for a bias term (resulting in a shape 46 × 20), we calculated for each site the mean absolute error between ${M}_{i,s}^{pred}$ and ${M}_{i,s}^{o}$ and divided this value by the standard deviation of each site. To avoid overfitting, leave-one-out cross-validation was used to build a separate model for each animal that is trained on all of the remaining data once that sample has been excluded. We kept those sites with MAE/σ < 0.50, resulting in 11,028 out of the original 284,860 sites deemed to be especially well predicted by our model.

We then reconstructed our model for the newly reduced methylation matrix ${M}_{a,s}^{o}$ (46 animals × 11,028 sites) and the trait matrix ${T}_{a,t}$ (46 animals × 19 traits + 1 constant). We predicted the values of each trait as ${T}_{a,t}^{pred}{={{M}_{a,s}^{o} C}_{t,s}}^{+}$ once again utilizing leave-out-one cross-validation. The Spearman correlation between the actual values of each trait ${T}_{a,t}$ and the predicted values ${T}_{a,t}^{pred}$ was used to identify methylation-associated traits. Each value in ${C}_{t,s}$ represents the site-specific coefficient associated to each trait. Those traits for which the Spearman correlation between the model predictions and expected output fell below 0.60 were removed from the model, with every remaining trait being re-predicted following the same steps as before. This process was repeated until all remaining traits equaled or surpassed 0.60. This resulted in 6 traits being maintained. The trait “rank” and the strain associated traits were forcibly included in all steps of the model due to their significance as traits of interest in this study. Without this constraint, rank would be removed at the trait filtering step of the model (Spearman =  ~ 0.3), and one of the two strain-associated traits would be removed in the collinearity phase.

Next, to select those sites whose methylation level is influenced by each trait, we estimated the significance of each coefficient in the model. For each of the remaining 11,028 sites we used multiple linear regression to analyse the relationship between the methylation level at that site and the methylation-associated traits:

$${y}_{i}= {\beta }_{0}+{\beta }_{1}{x}_{i1}+{\beta }_{2}{x}_{i2}+\dots +{\beta }_{p}{x}_{ip}+ \varepsilon$$

Here, ${y}_{i}$ is the dependent variable, e.g. the methylation status at site *i,* ${x}_{i}$ are the explanatory variables, e.g. the methylation-associated traits, ${\beta }_{0}$ is the y-intercept (constant term = 1), ${\beta }_{p}$ is the slope coefficient for each explanatory variable, and $\varepsilon$ is the model’s error term. Model p-values were adjusted for multiple hypothesis testing using the Benjamini & Hochberg method (*α* = 0.1) by trait.

### Model 2: Neural Network and Elastic Net Regression

We constructed a neural network using PyTorch (https://pytorch.org/), trained on cohort B, with timepoint information as well as trait information as input parameters. Each input vector corresponds to a specific animal/timepoint combination, resulting in 3,390 total input vectors across 345 animals, with the model being trained to predict the time, in weeks, a given animal is from death. The model consisted of an input layer, two hidden layers of 70 nodes each, and a single-node output layer representing the model’s prediction.

The input data was normalized via Z-score normalization with the exception of the strain, rank, and timepoint parameters. The model was trained to calculate the time until death value (time at death – current age) of a given sample. The forward propagation of the neural network for a single sample can be modelled as shown:

$$p=param[1, 2, \dots , n]$$

$${x}_{i,1}=max\left(0, {\sum }_{i=1}^{{n}_{parameters}} \left({{p}_{i}w}_{i,0}\right)+{b}_{i,1}\right)$$

$${x}_{i,2}= max\left(0, {\sum }_{i=1}^{n=70} \left({{x}_{i,1}w}_{i,1}\right)+{b}_{i,2}\right)$$

$${D}^{pred}={\sum }_{i=1}^{n=70} \left({{x}_{i,2}w}_{i,2}\right)+b_{3}$$

$$AE=|{D}^{pred}-D|$$

${p}_{i}$ represents the value of parameter $i$, ${x}_{i,n}$ represents the value of node *i* within layer *n*. ${w}_{i,n}$ and ${b}_{i,n}$ are the unique weight and bias values associated with node ${x}_{i,n}$. Note that for any node ${x}_{i,n}$ there exist *i* associated weight values and 1 associated bias value, all of which are unique. $p,$ ${x}_{1}$, ${x}_{2}$, and ${D}^{pred}$ represent the input parameters, hidden layer 1, hidden layer 2, and output layer respectively.

${D}^{pred}$ is the predicted time until death whereas $D$ is the actual time until death. We used a batch size of 32, with epoch size determined by dividing the total number of samples by the number of batches, rounded up. This resulted in ~ 86 epochs for each iteration of the validation process and 106 epochs when training the full model. Backpropagation was conducted using the Adam optimizer, with the initial learning rate set to 0.001. Samples were grouped such that all inputs associated with a given animal would be fully present within a single set (i.e. all timepoints associated with mouse x were used exclusively for either training or validation) to prevent data leakage. Mean absolute error (MAE) was used for loss calculation. To determine the accuracy of the model we used fivefold cross-validation, calculating the average loss value across all validation sets, resulting in an average loss of ~ 15.5.

This process was also performed on modified versions of the model. To determine the significance of each parameter in supporting the accuracy of the model, a single input parameter was excluded, for each input parameter, before assessment of model accuracy was performed using five-fold cross validation. To see if this significance changed when timepoint information was not present, this process was repeated once more but with timepoint information excluded entirely within every iteration. To further elucidate feature significance, we next retrained the model using all of the available parameters, then observed model accuracy given a deficient testing set. For each parameter within the testing set a single parameter was scrambled randomly, having its values replaced by a random, normal distribution of values. This process was repeated twice, first as described, and second with the additional scrambling of timepoint information. Tukey-pairwise comparisons were conducted using the distribution of validation losses for each model, with the base model being used as the control distribution. For the model variants which had two target parameters, the model with just timepoint as its target parameter was used as the control. We then adjusted the p values these comparisons produced using the Benjamini & Hochberg method. We then retrained two parallel models on all of the data within cohort B, with one variant including the timepoint parameter and one omitting it.

After these models were trained using the data from cohort B, we applied these models to make predictions using the trait data of Cohort A, generating predicted time until death values for each mouse/timepoint combination. The predictions generated by the model trained without timepoint information were used for validation purposes then discarded. The remaining values were then averaged with weight to derive a single value per animal, as well as to minimize noise due to model inaccuracy, with predictions being progressively more strongly weighted based on the input vector’s associated time point (week 12 = 0.02, week 16 = 0.05, month 6 = 0.08, month 8 = 0.11, month 10 = 0.14, month 12 = 0.17, month 14 = 0.20, month 16 = 0.23). Because some parameters possessed more timepoints than others, only those timepoints which recurred across all traits were considered. An increasing weight schema was chosen due to the collection of methylation data occurring at M17, with the last recorded trait information being collected at M16. We then grouped time until death predictions by rank, strain, and rank/strain combination, determining significance via Kendall coefficient (due to the small sample size).

To determine which sites could be associated with aging, we next utilized elastic net regression. The time until death predictions served as the dependent variable, while the methylation scores of the 284,860 sites served as the independent variables. The model hyperparameters were optimized using a combination of scikit-learn’s GridSearchCV, ElasticNet, and KFold classes, with fivefold cross validation being used to assess MAE post-hyperparameter (*α* and L1) optimization. The MAE was identified as 5.39, with the optimal α and L1 values both being identified as 0.1. This process resulted in 9,364 sites being maintained, with the remaining sites being assigned coefficients of 0. After converting all of the sites from mm39 to mm10 for further analysis, 9,363 sites remained.

We then combined the results of these two models by intersecting the set of sites selected via elastic net regression, with those sites associated with both blood glucose and strain, for both of these categories’ associated traits (i.e. the combination of M14 and M16 post-stress blood glucose and separately the combination of the strain-associated traits), to identify well-described SES-associated sites. M6 post-stress blood glucose and rank were excluded from analysis because they were not significantly associated with any sites. To help explain the over-representation of month 14 blood glucose compared to month 16 within the intersected set, we then averaged the blood glucose values for each of these timepoints by rank/strain combination and compared variances across and within groups for each timepoint.

### Functional enrichment analysis

The GREAT tool (indirectly via the greatbrowser module) was used to identify genes associated with each CpG site, and the Cistrome Toolkit was used to identify genes and transcription factors associated with the cumulative set of CpG sites (top 1000, ranked by AdjP value, excluding any AdjP > 0.01). For the Cistrome plots, the 1000 largest sites, ranked by coefficient magnitude, were used for the model 1 plots, whereas all sites not equal to 0 were used for the model 2 plot (~ 9500).

## Results

### Description of the population and experimental variables

We sought to examine the relationship between stress, healthspan, lifespan and DNAm. To this end we examined two cohorts of a large dataset derived from mice exposed to lifelong psychosocial stress (as detailed in [6]). Starting at 12 weeks of age dyads of male mice underwent a 4-week chronic psychosocial stress protocol, consisting of daily brief direct exposures to physical interaction/aggression culminating in social defeat of one of the two mice, followed by 24 h sensory housing. After this phase mice entered an aging phase and continued the study in co-housing and sensory contact conditions (Supplementary Fig. 1). Healthspan data including food intake, body weight, body composition and plasma glucose levels were collected regularly from both cohorts.

**Cohort A:** Healthspan traits were collected from the 12 th week of age through 16 months of age, and DNA methylation data were collected from the liver post-sacrifice at 17 months: n = 18 C57BL/6J, n = 12 CD1 and n = 16 Sv129Ev, that were also categorized by social rank as dominant (N = 17, n = 5 C57BL/6J, n = 8 CD1, and n = 4 Sv129Ev), subordinate (N = 17, n = 8 C57BL/6J, n = 0 CD1, and n = 9 Sv129Ev) or undefined, thus classified due to the lack of a stable hierarchy (N = 12, n = 5 C57BL/6J, n = 4 CD1, and n = 3 Sv129Ev).

**Cohort B:** Cohort B (N = 345) was followed to detect healthspan during the lifecourse and survival. Cohort B was composed by 172 C57BL/6J (56 dominant, 60 subordinate, 56 undefined), 86 CD1 (52 dominant, 13 subordinate, 21 undefined) and 87 Sv129Ev (8 dominant, 42 subordinate, and 37 undefined).

### A multivariate multiple regression model to identify blood glucose and mouse strain as major traits associated with DNA methylation

**Fig. 1:**
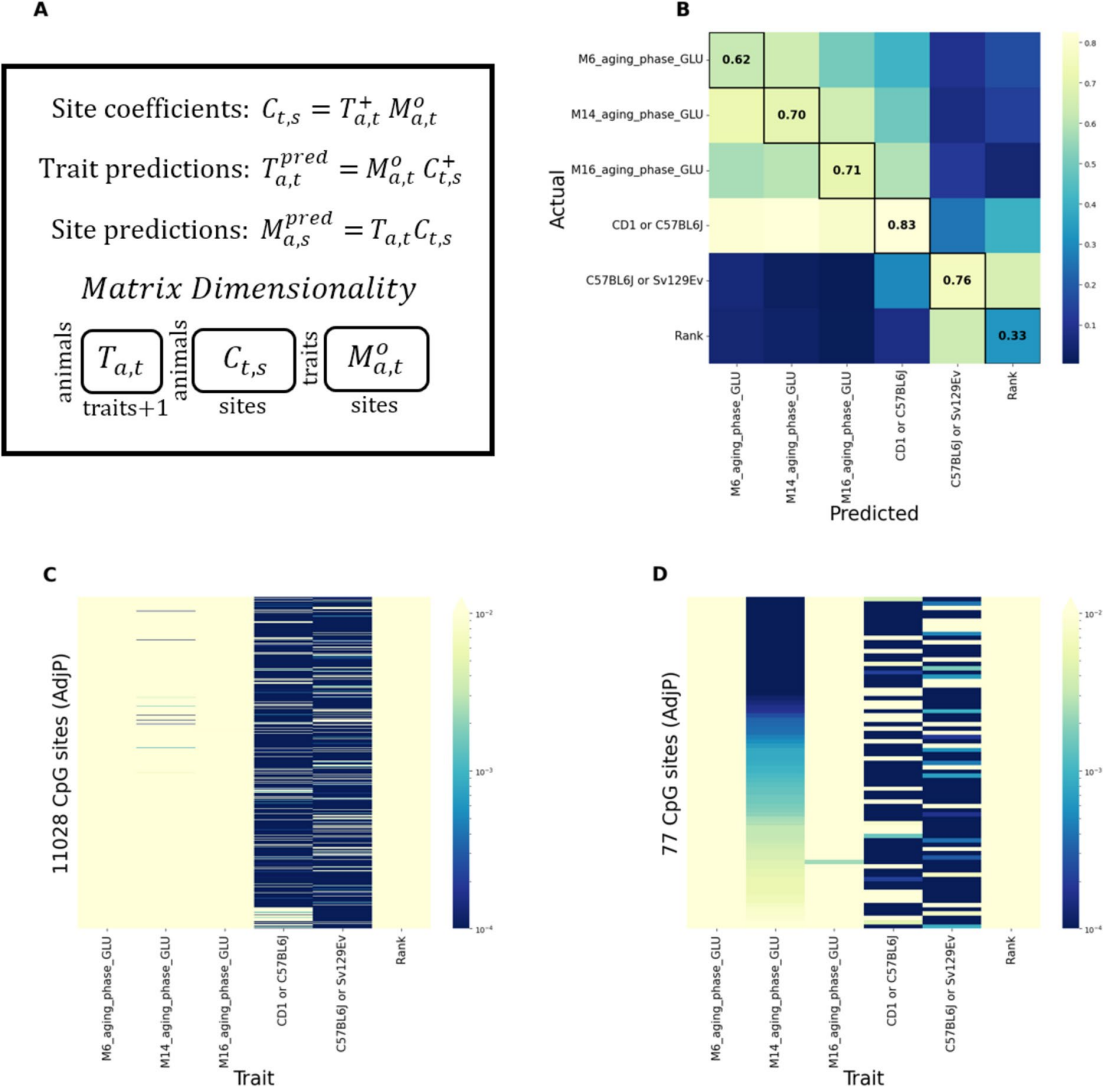
MMR and MLR results. (**A**) The equations used in the MMR model and the dimensions of the utilized matrices. (**B**) The Spearman correlation between the MMR model predictions and the actual values. (**C**) The results of the MLR analysis for each trait, with the AdjP (log) for each site/trait combination being shown. (**D**) The results of the MLR analysis, highlighting all significant sites (AdjP < 0.01) for the M14_aging_phase_GLU model specifically

In the first analysis we constructed a bi-partite model utilizing multivariate multiple regression (MMR) followed by several independent multiple linear regression (MLR) models to study the relationship between DNAm and healthspan traits (Fig. 1A). The overall control flow of the data analysis is shown in Supplementary Fig. 2, with the division of mice cohorts being shown in Supplementary Fig. 1. Our analysis sought to identify the influence of diverse traits on the methylation of CpGs measured in Cohort A, with “traits” herein referring to those biomarkers assessed via direct phenotypization.

After preprocessing the data by removing highly colinear traits (Spearman > 0.7) (Supplementary Fig. 3), resulting in 19 traits being maintained, we utilized MMR (Fig. 1A) to identify methylation sites predicted with high accuracy and with a high degree of variance across individuals (MAE/σ < 0.5), resulting in 11,028 sites being maintained. To determine which of these traits were most informative to the model, we developed an inverse model to predict traits from methylation using these sites (Fig. 1A). This approach allowed us to identify highly predictable traits (Spearman > 0.6) which are thus more likely to significantly influence methylation. This led to 6 traits being selected, most of which were measurements of blood glucose: M6_aging_phase_GLU (blood glucose at 6 months of age, during the aging phase), M14_aging_phase_GLU (blood glucose at 14 months of age, during the aging phase), M16_aging_phase_GLU (blood glucose at 16 months of age, during the aging phase), Rank (relative dominance to other animal), and strain (which was encoded across two traits as C57Bl/6J or Sv129Ev and CD1 or C57Bl/6J) (Fig. 1B). The trait “rank” as well as the strain-associated traits were forcibly included in all steps of the model due to their significance for this study.

**Fig. 2:**
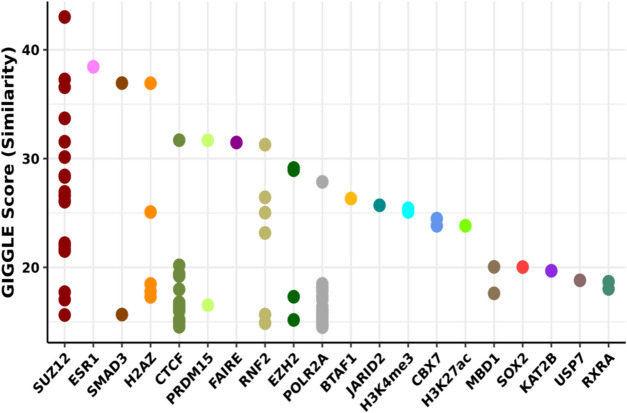
Cistrome plot for blood glucose. Every site selected (AdjP <  = 0.01) by the multiple linear regression model, for all blood-glucose associated traits, was run collectively through the Cistrome Toolkit. See Supplementary Fig. 4 for more information

Having narrowed down those sites and traits best predicted by our model, we then developed independent MLR models for each site to generate P values for each trait/site combination, and adjusted these values across each trait using the Benjamini & Hochberg method. This allowed us to identify those sites significantly (AdjP < 0.01) associated with each given trait (Fig. 1C). We identified 13 sites associated with M16_aging_phase_GLU, 77 sites associated with M14_aging_phase_GLU (with 89 sites being associated with either timepoint, indicating very little overlap between the two sets (Fig. 1D)), and 11,023 sites associated with strain (either of the two encoded traits). These results cumulatively indicate that strain and blood glucose are both well predicted using methylation scores, with strain being highly significant across many sites. We then converted the assembly of sites from mm39 to mm10 for further analyses, which resulted in 3 sites being dropped, and combined the related sets into “blood glucose” and “strain” respectively. To determine whether these sites share common factors we analyzed them using the Cistrome ToolKit, generating lists of transcription factors whose binding significantly overlaps the top 1000 (ranked by coefficient magnitude) CpG sites associated with each trait (Fig. 2, Supplementary Fig. 4). For example, SUZ12 was strongly identified within the blood glucose set across many biosamples, while also making several (less correlated) appearances within the strain set, with the opposite being true for RNF2, EZH2, and POLR2 A across the strain and blood glucose sets (Fig. 2, Supplementary Fig. 4).

### Neural network analysis to identify traits associated with mortality risk

**Fig. 3:**
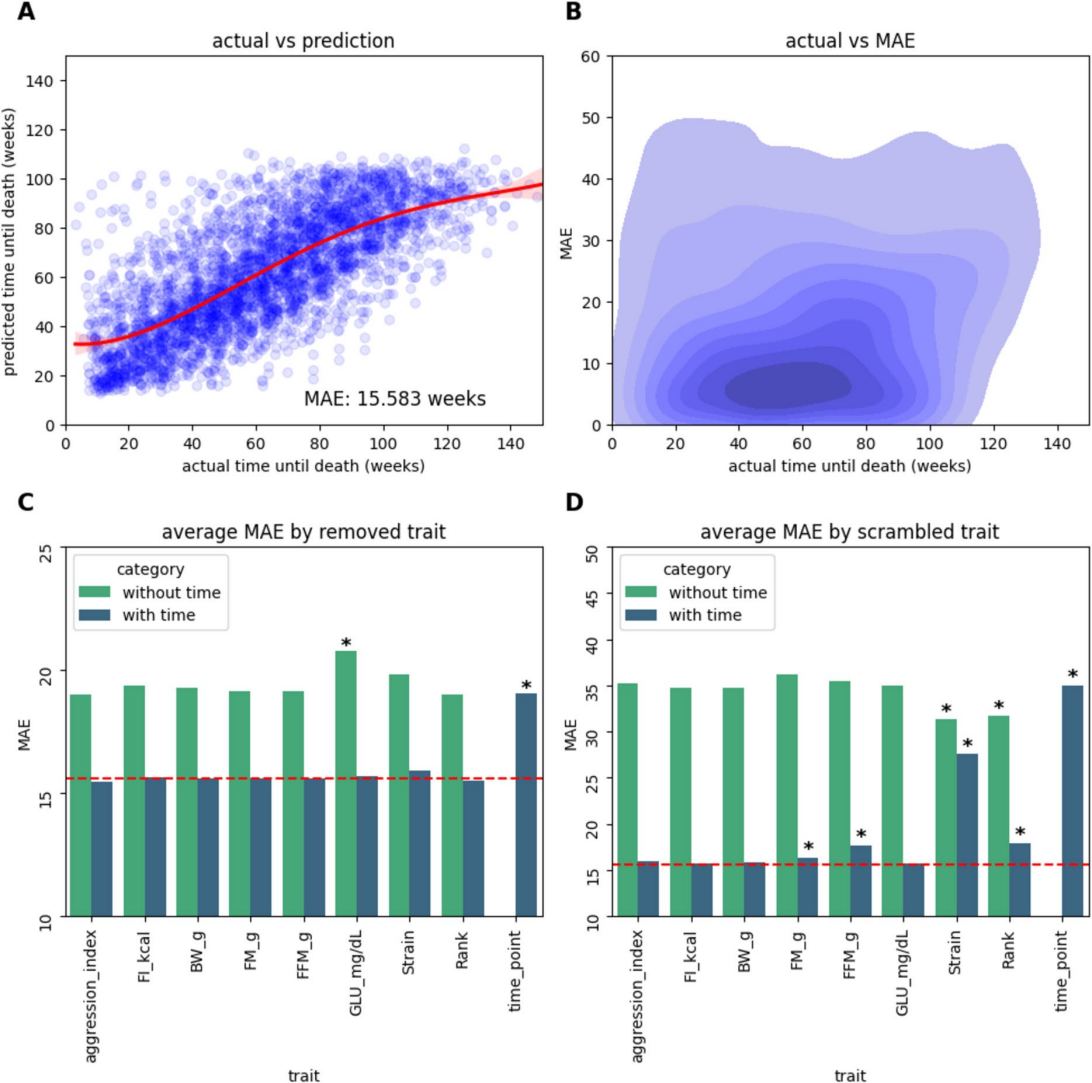
Accuracy of the neural network model. (**A**) Model approximations versus actual time until death. (**B**) Distribution of the loss of model predictions with respect to the actual time until death. (**C**) Loss values corresponding to the model with the exclusion of a given parameter. Each parameter was removed both with and without the additional removal of timepoint. The “with time” category refers to the removal of only the parameter, whereas the “without time” category refers to the removal of both the target parameter and timepoint. FI_kcal = food intake (kcal); BW_g = body weight (g); FM_G = fat mass (g); FFM_g = fat-free mass (g); GLU_mg/dL = plasma glucose (mg/dL). * indicates significance. (**D**) Loss values corresponding to the model given the scrambling of the target parameter(s). The model was trained a single time, with scrambling occurring during the validation phase. * indicates significance

Our next step was to use the data from Cohort B, consisting of 345 mice, for whom time at death and trait data were recorded, to train a neural network model to predict mortality risk. The analysis of this network was intended to provide insight into which traits were most predictive of mortality as well as the relationship between these traits. The data from 3,390 mouse/timepoint combinations were used as feature vectors, including both the trait data as well as the timepoint when the data was collected, with the corresponding time until death (time at death – current age) of each combination as the response variable that the model was trained to predict. The model was validated using a fivefold cross validation schema, with the mean absolute error (MAE) being used for loss calculation (Fig. 3A-B). We observed that the accuracy of the model was at its best when predicting values clustered around 60, representing 60 weeks until death, which is not surprising given that the median time until death value for the training data was approximately 61 weeks. The average loss of the model was 15.5, i.e. an average inaccuracy of approximately 15.5 weeks.

To measure the significance of each parameter on the model’s accuracy, we repeated this procedure with the removal of a single parameter during training, iterating through all available parameters. Then, to determine which parameters the model was reliant upon when deprived of temporal information, we once again repeated this procedure (the exclusion of a single parameter) in addition to the constant exclusion of timepoint information (Fig. 3C). Next, as an alternative method aimed at further clarifying feature significance, we retrained the model fully, using the entirety of the training set, and observed the loss produced by the model when a given feature was scrambled during validation. This was done for all the features twice, once with only the target feature being modified, and once with timepoint additionally being scrambled (Fig. 3D). Tukey–Kramer pairwise comparisons were conducted to assess significance, then adjusted using the Benjamini–Hochberg method, with the unmodified, timepoint-removed, and timepoint-scrambled models respectively serving as the control distributions for the altered models. We observed that, within the removed group (Fig. 3C), both timepoint (AdjP <  < 0.001) and the combination of timepoint and blood glucose (AdjP <  < 0.001) significantly altered model accuracy, whereas in the scrambled group (Fig. 3D), fat mass (AdjP < 0.05), fat-free mass (AdjP <  < 0.001), timepoint (AdjP <  < 0.001), strain (AdjP <  < 0.001), rank (AdjP <  < 0.001), the removal of both timepoint and strain (AdjP <  < 0.001), and the removal of both timepoint and rank (AdjP <  < 0.001) were all found to induce significant changes in model accuracy. Both methods identified timepoint as significantly underlying model accuracy, as we would expect, but it is interesting to observe that no other overlap was found across our assessments of model accuracy. Cumulatively, these results indicate that rank, strain, fat-free mass, fat-mass, blood glucose and timepoint most strongly underlie the model’s ability to assess mortality risk.

### Elastic net regression to identify sites associated with aging

**Fig. 4:**
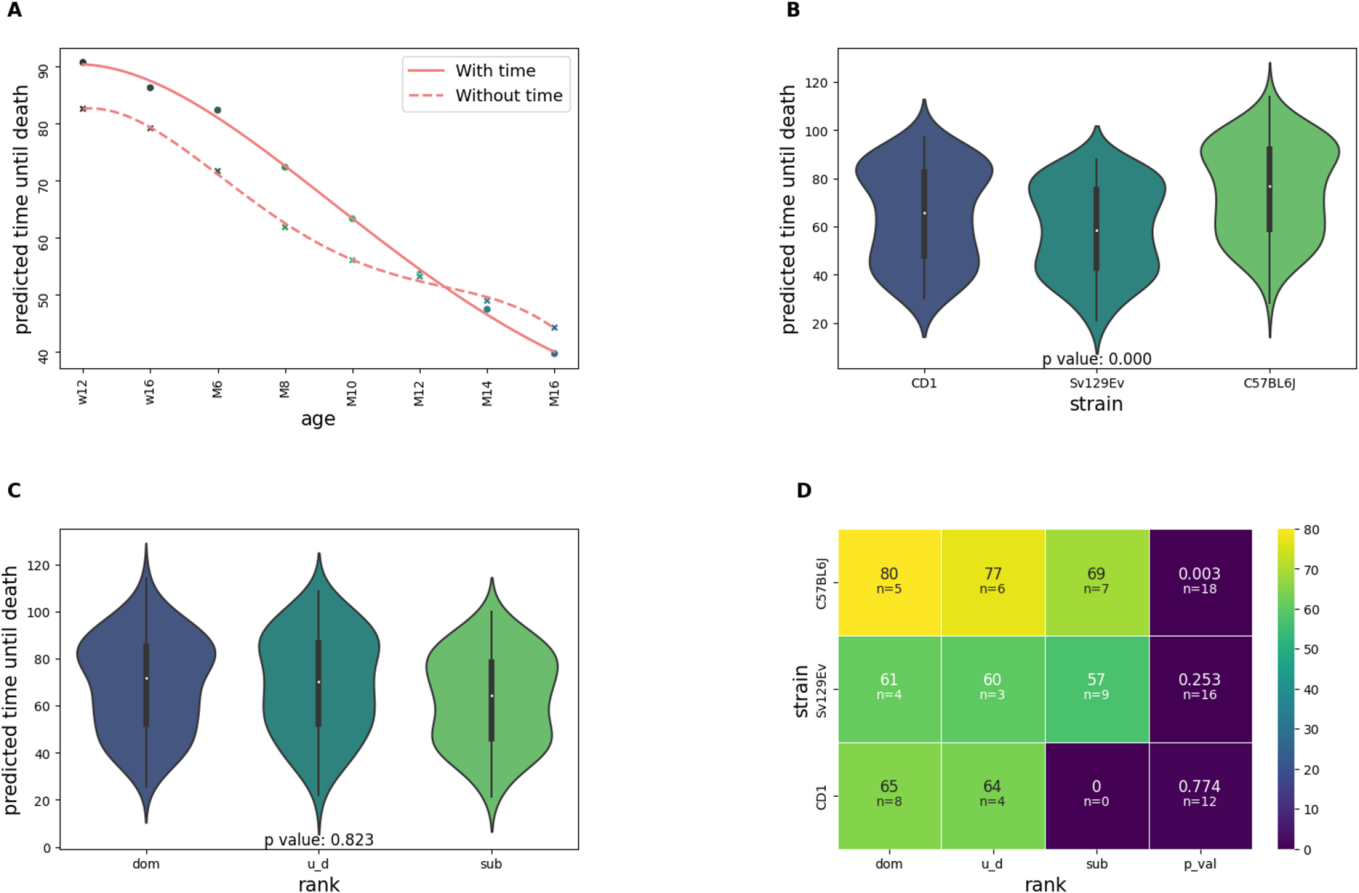
“Predicted time until death” values outputted by the neural network. (**A**) Average value by timepoint for the full model as well as for the parallel model trained without timepoint. The filled line represents the full model, whereas the dashed line represents the model trained without timepoint (w = week, M = month). (**B**) Distribution by animal strain. (**C**) Distribution by social rank. (**D**) Distribution by rank/strain combination, and P-value for the relationship between rank and strain by strain. The average prediction (expressed in weeks until death) and number of animals for each category is shown within each block. As there are no sub/CD1 animals, the value shown is 0/n = 0

Our next step was to apply this model to Cohort A for which DNAm was available and generate corresponding time until death predictions for each mouse/timepoint combination. Natural time until death in this case could not be measured as all of the mice were sacrificed. This procedure allowed us to assess the relationship between Cohort A’s DNAm data and mortality risk (predicted remaining lifespan), something we could not do using Cohort B due to a lack of DNAm data (Supplementary Fig. 1). We grouped these predictions by timepoint, to verify the model’s accuracy, as well as by rank, strain, and rank/strain combination to identify group-wide trends (Fig. 4). As shown in Fig. 4A, the model was correctly able to predict that animals assessed at later timepoints were, on average, closer to death than those assessed at earlier timepoints, showing general trend recognition. This was observed to be the case regardless of whether the model was trained using timepoint or not (Fig. 4A). With regards to strain, C57BL/6J, CD1 and Sv129Ev were each associated with increasing mortality risks respectively, establishing the role of strain as being statistically significant in influencing time until death (P < 0.001, Fig. 4B) and in agreement with previous data on the actual mortality of these strains exposed to LCPS [6]. With regards to rank, the lower in rank an animal was [subordinate (sub) < undefined (u_d) < dominant (dom)], the closer to death the animal was predicted to be, albeit without reaching a significant level (P = 0.82, Fig. 4C). This relationship was better exposed when rank and strain were considered in conjunction (Fig. 4D), with C57BL/6J exclusively showing a significantly decreased time until death in lower rank animals (P < 0.005). Interestingly, the predictive ability of the current mathematical model mirrors the observed reduced survival of C57BL/6J mice classified as subordinate [18].

The time until death predictions for each associated timepoint/animal combination generated by the primary model were then averaged, across each animal, with weights varying from 0.02 to 0.23, increasing by 0.03 with each following timepoint (week 12 = 0.02, week 16 = 0.05, month 6 = 0.08, etc.) so that each mouse would have a single associated value, as well as to minimize noise due to model inaccuracy. An increasing weight schema was chosen due to the collection of DNAm data occurring at 17 months of age, with the last recorded trait values being collected at 16 months of age, in Cohort A.

**Fig. 5:**
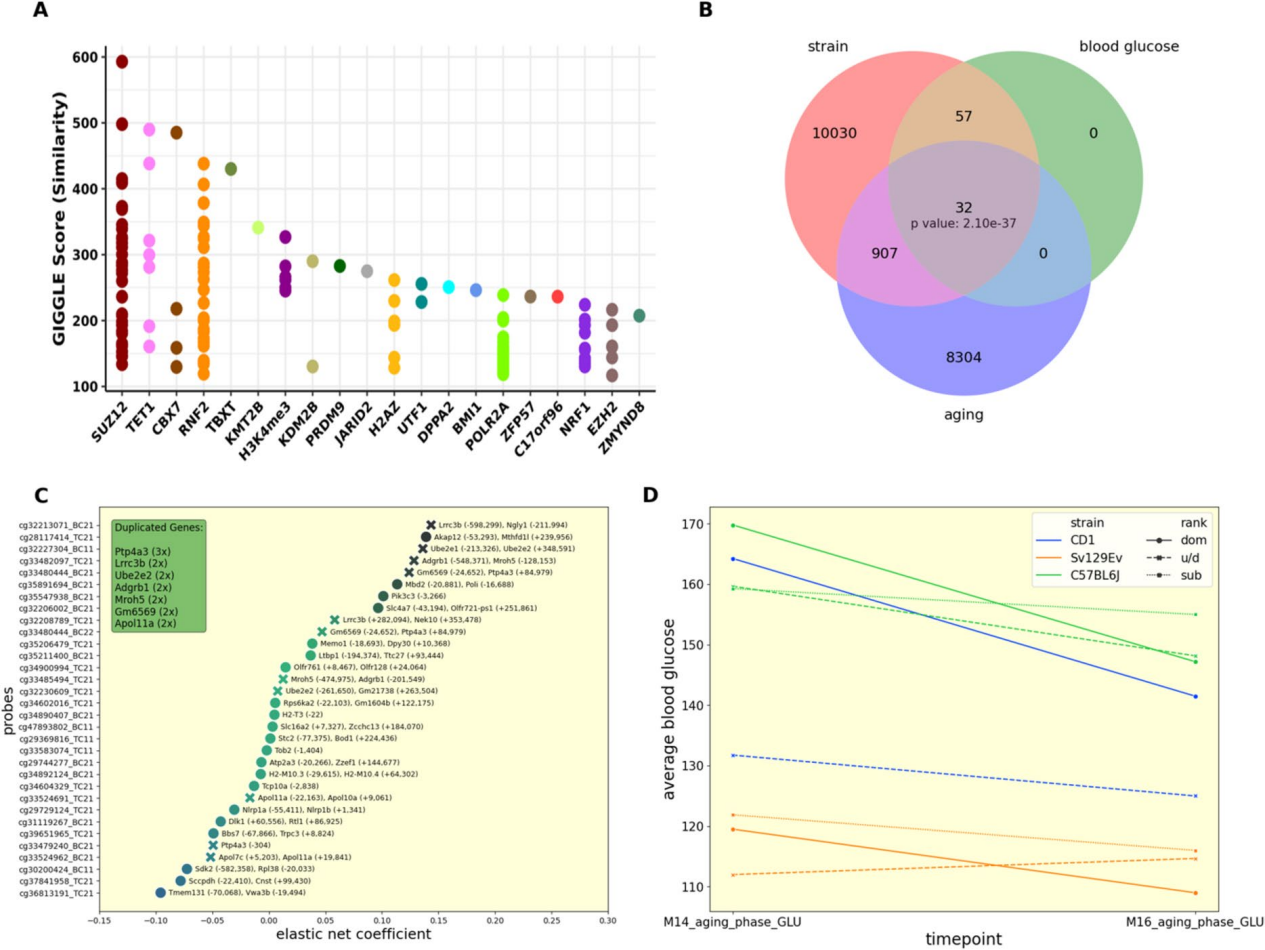
Associations between significant CpG sites, genes, and transcription factors. (**A**) Every site selected by the elastic net model run collectively through the Cistrome Toolkit. See Supplementary Fig. 4 for more information. (**B**) Venn diagram depicting the overlap between the blood glucose, strain, and aging (mortality risk) sets. The p value is also shown for the intersection of all 3 sets. (**C**) The genes associated with the sites overlapped between the blood glucose, strain, and aging sets, and a table indicating how many times repeated genes were observed. (**D**) The blood glucose values at months 14 and 16 with respect to rank and strain. Strain is differentiated by color while rank is differentiated by line fill

We then utilized elastic net regression to identify those methylation sites which are most strongly associated with the averaged time until death predictions, informed by the same rationale guiding the construction of epigenetic clocks [36]. To determine the accuracy of the model we utilized fivefold cross validation, with the model MAE being identified as 5.39 post-hyperparameter (*α* and L1) optimization. We then retrained the regression model using all the available animals. This resulted in 9,364 sites being selected out of the original 284,860, reduced to 9,363 after conversion to mm10, all of which were subsequently ran through the Cistrome Toolkit (Fig. 5A). From this analysis we note that SUZ12 and RNF2 both showed a high degree of correlation across numerous biosamples. This aligns with the results of the previous Cistrome analysis conducted trait-wise, which identified the same genes as being associated with blood glucose (Fig. 2).

We next considered the intersection between the sites selected across both models. Given that the feature evaluation indicated a significant role for both blood glucose (Fig. 3C) and strain (Supplementary Fig. 4) in the determination of the neural network’s accuracy, we opted to overlap those sites associated with blood glucose and strain by model 1, with those sites associated with aging by model 2. Despite the fact that these three site subsets account for ~ 4% (11,026/284,860), ~ 0.03% (89/284,860), and ~ 3% (9,363/284,860) of the total site set for the strain-associated sites, blood glucose-associated sites, and aging-associated sites respectively, 32 sites (~ 36% of the blood glucose set) were concurrently observed within all three sets. The overlap of these sets is shown in Fig. 5B (p <  < 0.001), with the gene associations of these sites shown in Fig. 5C. In particular, Ptp4a3, Lrrc3b, Adgrb1, Mron5, and Gm6549 were identified as genes associated with multiple sites (Fig. 5C; see legend), and therefore represent possible targets at the intersection of glucose, strain and survival. To further elucidate these results, we next considered whether blood glucose at 14 months or blood glucose at 16 months contributed more to those sites represented within the intersection set. We observed that, of the 32 selected sites, 30 were present within the month 14 blood glucose set exclusively, with 1 being present in both (cg35211400_BC21). To help determine why this might be, we considered the average blood glucose values for both timepoints (M14 and M16) for each rank/strain combination (Fig. 5D). ANOVA analyses of the blood glucose levels for months 14 and 16 with respect to each rank/strain combination revealed that both months were significantly different across each rank/strain combination. Month 16 was identified as being more strongly clustered by group (F = 3.02, p = 0.013) compared to month 14 (F = 2.6, p = 0.025), which may indicate that the month 14 blood glucose values better capture the individual nuances of the phenotype than the month 16 values.

## Discussion

Here, we utilized a combination of two computational models to identify variations in and across mortality risk, phenotype, and DNA methylation, in the context of chronic psychosocial stress. This holistic approach allowed us to identify those loci and metabolic traits which rest at the intersection of these components, and thus at the intersection of the biologic substrates of chronic psychosocial stress.

Specifically, model 1 allowed us to predict DNAm values using a combination of traits. Among these, the most compelling association was found for blood glucose which was more strongly associated with DNAm than social rank [6, 37–39]. This may be due to the origin of the utilized DNAm data from liver tissue samples, an organ primarily involved in the regulation of glycemia. It may also be that psychosocial stress impacts the epigenetics underlying blood glucose to a greater extent than other traits, though the results offered here are insufficient to bolster this claim. Glucose homeostasis has been proposed to be central for healthspan and lifespan in humans [40, 41], with elevated glycemic biomarkers showing the most detrimental effects [42]. Studies in mice show decreasing levels of blood glucose with age, though with a different pattern than in humans [39, 43–45]. This supports the centrality of glycemic control while also highlighting distinct glucoregulatory patterns across species, and therefore the need for a deeper mechanistic understanding to inform translational studies [39].

Strain was also associated with various CpG sites, which is unsurprising given the close relationship between epigenetic and genetic variations [46].

In an effort to further draw comparisons between those identified traits and genes in the context of mortality risk, and in consideration of the technical and executive constraints in carrying out lifelong stress studies in rodents, model 2 was implemented as a more biologically feasible and broadly interpretable approach to epigenetic clock construction. This model in turn highlighted a wide variety of traits and CpG sites significantly underlying mortality risk. These included strain, rank, blood glucose, fat-free mass, and fat-mass, all of which are consistent with prior research [6]. We then considered the results of models 1 and 2 with respect to one another and observed that the intersection between those CpG sites associated with strain, blood glucose, and mortality risk was much more strongly informed by the month 14 blood glucose sites than those associated with month 16. This is in spite of the larger gap in time between these earlier measurements and the collection of methylation data at month 17. Statistical analyses identified a higher degree of clustering by rank/strain combination for the month 16 blood glucose data, which may indicate that individual variances in aging-associated methylation are not as well captured by later glucose timepoints. These results may indicate that certain transient changes in blood glucose levels are capable of predicting long-term downstream methylome aberrations in ways not sufficiently captured when considering only social rank or animal strain.

In consideration of those genes identified by this intersection, Ptp4a3 was selected across more sites (3/32) than any other gene within the tri-intersected set. Ptp4a3 has previously been identified as a protein-tyrosine phosphatase associated with the plasma membrane [47], specifically within the blood brain barrier. This protein is also a potential biomarker for Alzheimer's disease (AD) [48]. Notably, AD is strongly associated with metabolic dysregulation including diabetes [49, 50], and we previously reported glucose intolerance [51] and increased protein biomarkers of AD in the LCPS model [52]. We simultaneously identified the genes Lrrc3b, Apol11a, Adgrb1, and Mroh5 across multiple sites. Lrrc3b is a tumour suppressant gene that has been associated with numerous cancers [53]. Apol11a is a gene linked to focal segmental glomerulosclerosis as well as schizophrenia [47]. Adgrb1 has been associated with social deficits and seizure susceptibility in mice [54], and Mroh5 has been associated with neutropenia [55]. Each of these genes provides us with a functional linkage between some disease state and chronic social stress.

These analyses also provided us with a variety of transcription factors which recurred across CpG sets, as an indication of how these sites generated downstream changes across the greater epigenetic landscape. Suz12, Rnf2, and Polr2 A were all found to be associated with all inputted site sets across numerous biosamples, with a strong association with strain, blood glucose levels, and mortality risk. Both Suz12 and Rnf2 have previously been identified as members of the polycomb repressive complex 2 (PRC2). These complexes have been observed to regulate a wide array of cell types, including pancreatic beta-cells responsible for the secretion of insulin [56] and, by extension, the modulation of blood glucose levels. Importantly, the targets of PRC2 are also known to strongly overlap with those CpGs known to gain methylation with age, to the point that epigenetic clock construction based upon this targeting has been proposed [57]. Suz12 has also been independently associated with varying methylation levels in the context of aging [58], whereas Rnf2 is known to be associated with ubiquitination in response to chronic glucose stress [59]. Polr2 A is known to encode for the largest subunit of RNA polymerase II [47]. Its regulation is crucially dependent on DNAm levels [60] that modulate its elongation and alternative splicing, with consequences associated to cellular senescence and aging [61, 62]. These results suggest that Polr2 A, Suz12 and Rnf2 may play a significant role in the acceleration of aging and the abnormal phenotypes observed due to chronic psychosocial stress. Cumulatively, these results demonstrate to us the power of diverse computational methods in providing a unified depiction of chronic psychosocial stress as a modifier of blood glucose, fat-mass, fat-free mass, rank, strain, mortality risk, and the epigenetic landscape of the individual. Notably, rank and strain emerge as key modifiers, with blood glucose serving as the most effective descriptor of the connection between liver DNAm and mortality risk.

### Limitations of the study and future direction

The primary limitation of this study is the lack of longitudinal epigenetic data from which to form an epigenetic clock, given the lack of applicability of other, pre-made epigenetic clocks to our study design. This limitation was in part addressed by developing model 2, and while this approach does offer some unique insights beyond those provided by a standard epigenetic clock, such as the consideration of actual mortality risk over age, and the ability to evaluate feature significance in model training, the success of this method is limited by the relatively small number of mice within in this study. The effect of the limited sample size on the model’s accuracy is twofold: firstly, through limiting the amount of data upon which the model was able to be trained, and secondly, through limiting the ways in which this data could be structured for efficient pattern recognition. Namely, we were unable to use those non-linear models typically associated with non-linear longitudinal data (LSTMs, transformers, etc.) as doing so would drastically decrease the number of available subjects (which is especially problematic given the large amount of data demanded by these models). Another limitation is that we used a novel method, but could not realistically perform independent validation due to the technical challenges that would be associated with this, of which the model was intended to circumvent (i.e. building an epigenetic clock). Finally, a limitation of this study which is common to many similar approaches is the so called “black box problem”, which describes the innate difficulty of determining how neural network models make their decisions. Because of this, it is difficult to identify those traits which most strongly contribute to model predictions and how these traits interact with one another. This was partially accounted for through the usage of two distinct feature identification methods, however the lack of overlap between the results of these two methods further highlights the difficulty of this problem. Future studies should aim to resolve the data-hungry problem, addressing both longitudinally recorded epigenetic data as well as feasibility aspects. Regardless, the present findings align with previously published research [6, 18], highlighting the robust application of computational techniques to biological research. These techniques effectively identify genes, traits, and transcription factors at the intersection of chronic psychosocial stress.

## Supplementary Information

Below is the link to the electronic supplementary material.

Supplementary file1 (DOCX 775 KB)
