# Supplementary material for: Epigenetic biomarkers of mortality risk in mice under chronic social stress
Source: GeroScience. 2025 Jun 9;48(1):1127–42. doi: 10.1007/s11357-025-01721-7 (PMC12972157; doi:10.1007/s11357-025-01721-7)
Supplement: Supplementary file 1 — Supplementary file1 (DOCX 775 KB) [file 11357_2025_1721_MOESM1_ESM.docx]

**Supplementary Figures**


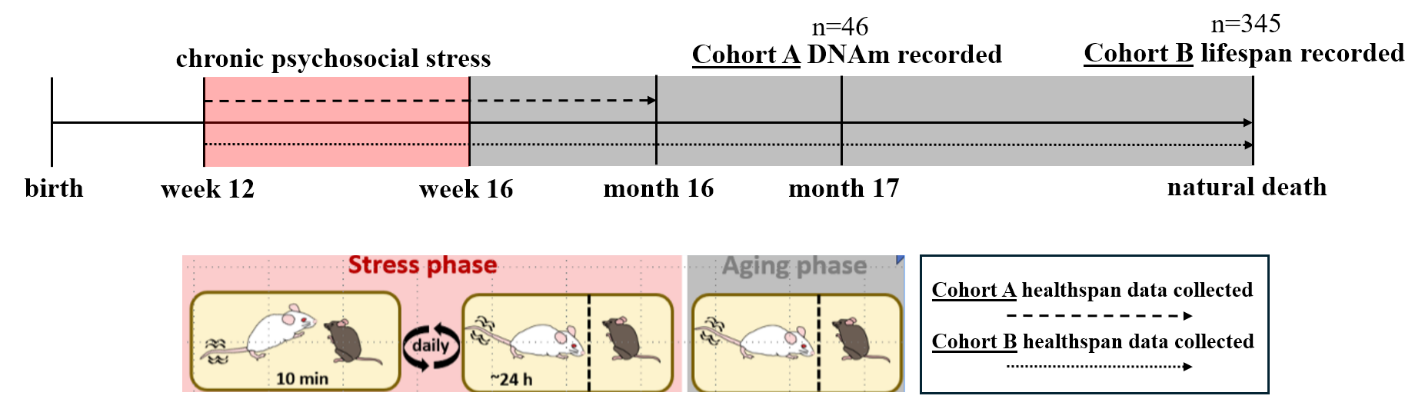


**Supplementary Figure 1. Overview of the experimental design.** Mice were exposed to a protocol of lifelong psychosocial stress. Starting from the age of 12 weeks, and ending at the age of 16 weeks (4 weeks total), mice were exposed to daily, brief (10 minute) social physical interaction/aggression, followed by sensory contact in co-housing. After these 4 weeks, mice were maintained in sensory contact co-housing, with trait information being collected at regular intervals. Two cohorts were derived from these mice, 46 who were sacrificed at 17 months of age, with their DNAm collected (Cohort A), and 345 who were allowed to age naturally, with their lifespan recorded (Cohort B).

**
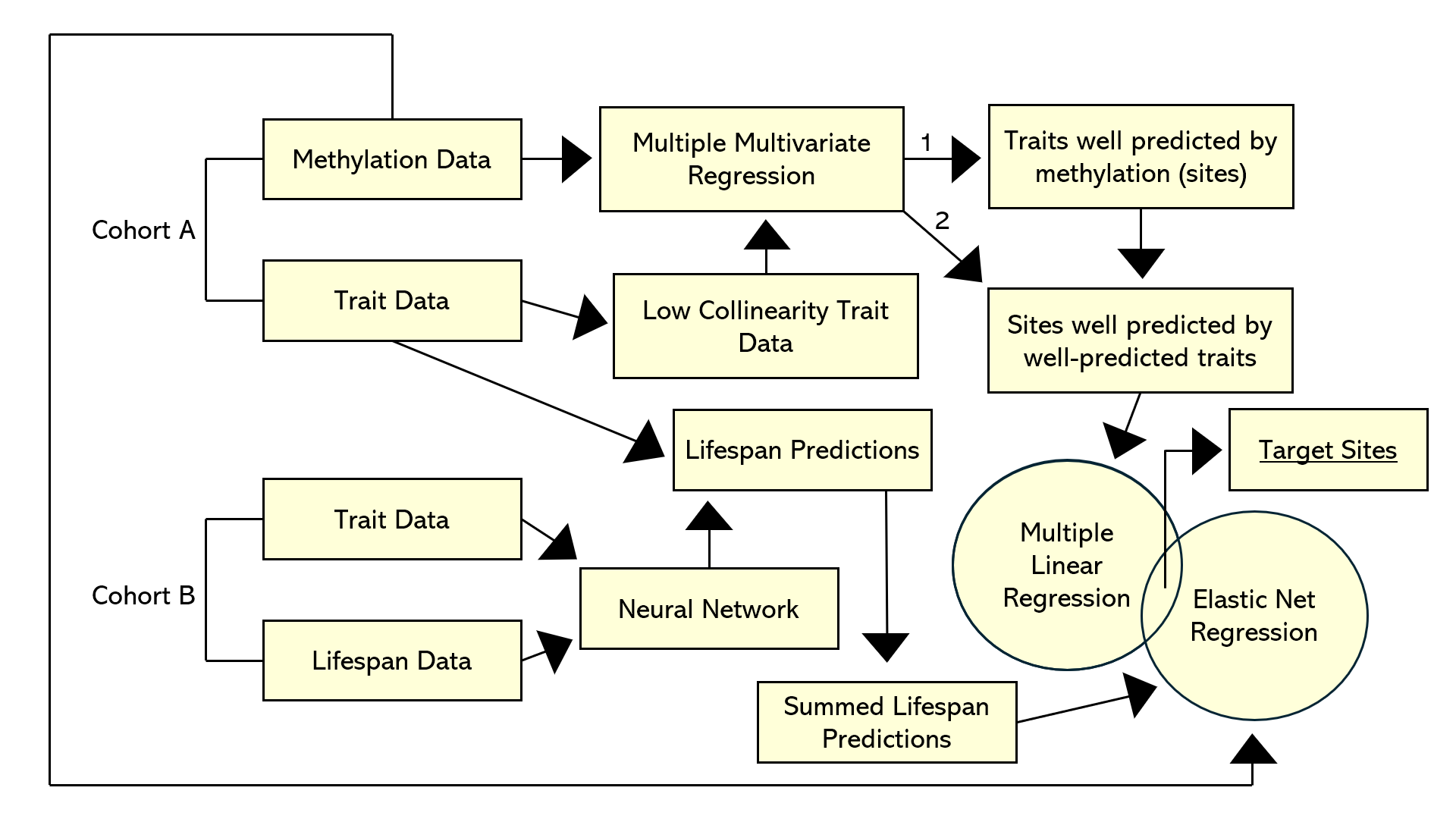
**

**Supplementary Figure 2. The overall control flow of the analysis.** The trait data from cohort A is filtered to remove collinearity before being used in conjunction with the methylation data from cohort A to establish an MMR model, from which those sites highly predictable using the pruned traits are determined. Next, those traits highly predictable using the pruned sites are ascertained, further pruning the traits. The two filtered datasets are then utilized in multiple linear regression, generating a set of significant sites for each trait. The trait data from cohort B is normalized before being used in conjunction with the timepoint data from cohort B to train a neural network, which is then applied to the trait data of cohort A, generating time until death predictions for each animal/timepoint combination. These values are then combined using a weighted average, and these averages are used in conjunction with the unfiltered methylation data from cohort A as part of an elastic net model. Elastic net regression identifies those sites which best describe the model’s predictions. These sites are then intersected with those sites identified previously by the multiple linear regression model.


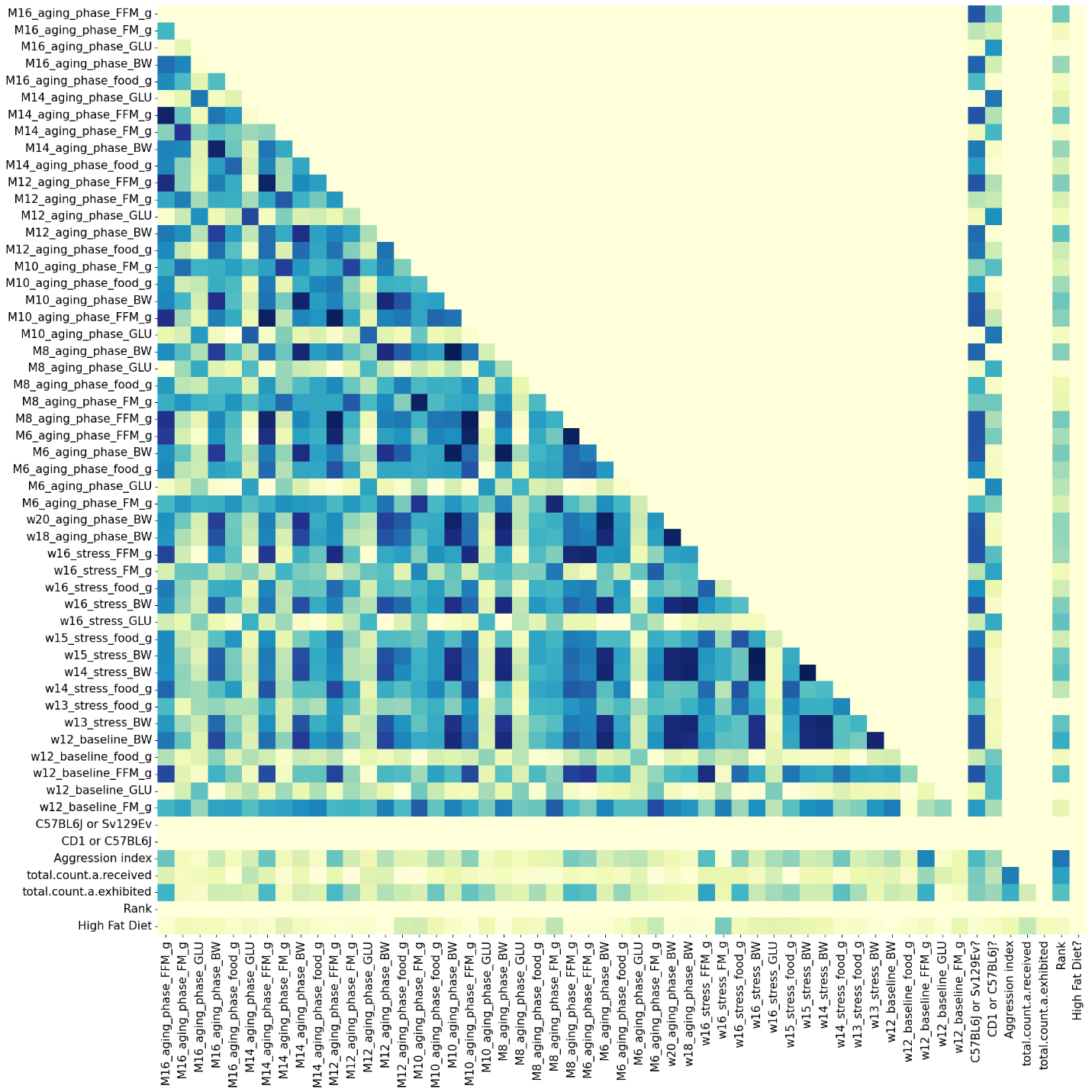


**Supplementary Figure 3. The spearman coefficient between each trait.** Traits were sorted by timepoint (descending) before being used to construct a matrix with dimensions 55x55, with each row *i* and column *j* representing the same trait, and with values representing the spearman coefficient of the intersection. Masking was applied to the diagonal, to ignore any self-correlations, and above the diagonal, to avoid repetitive information due to the symmetric nature of the matrix. To avoid removing the strain-associated traits or rank information while still allowing them to be involved in filtering, we reset the values of these columns, undoing any masking. We then fully masking their associated rows, so that they would not have any values greater than 0.7. We then removed every row which possessed any value greater than 0.7 to identify a set of traits with minimized collinearity.


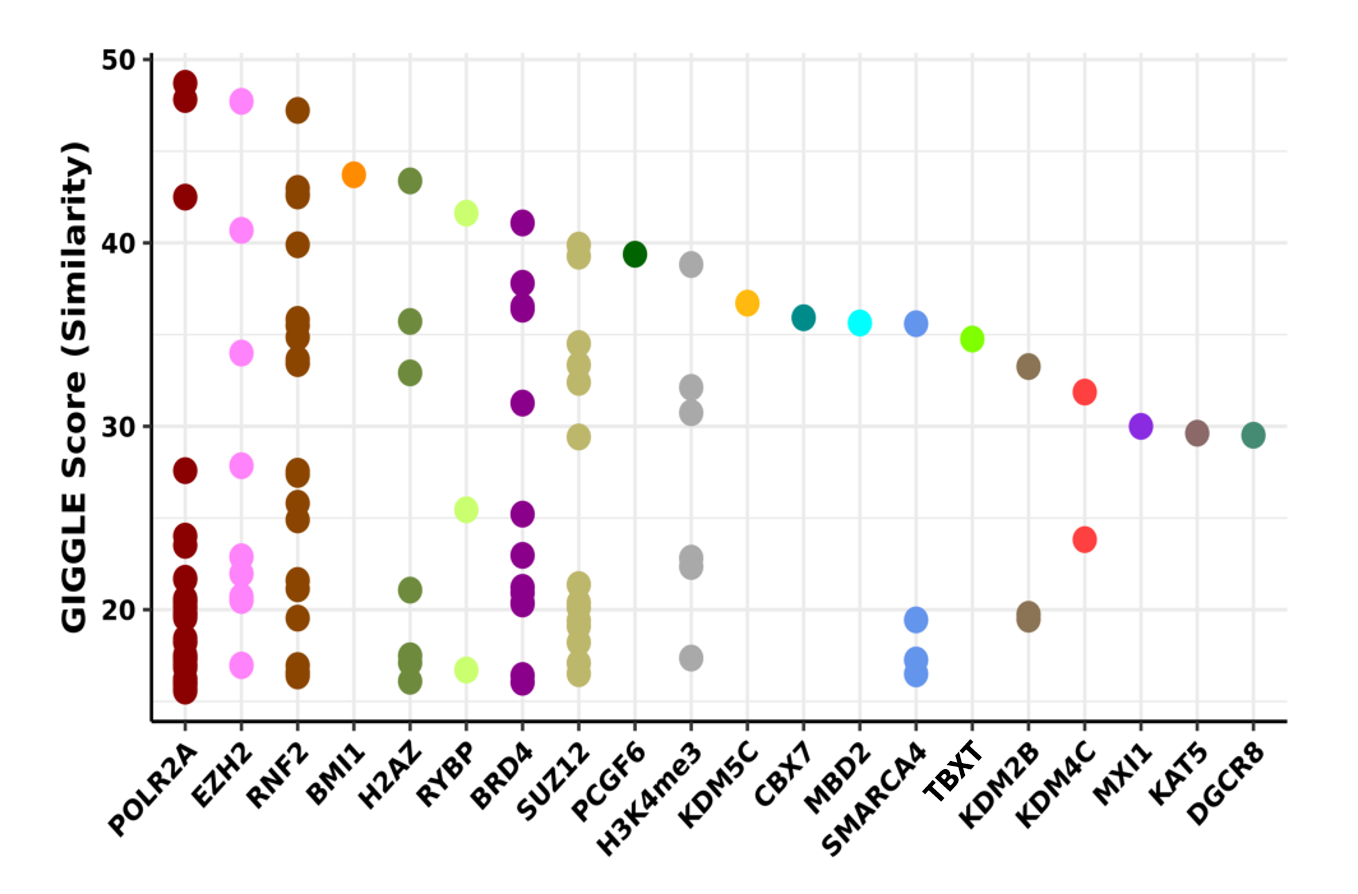


**Supplementary Figure 4.** The top 1000 strain-associated sites run collectively through the Cistrome Toolkit. Ranking was determined by coefficient absolute value, averaging the coefficients together or selecting the available coefficient if a probe was not selected for both associated traits. The Cistrome Toolkit outputs tables and plots indicating the similarity between the inputted site set and those previously identified, grouped on the x axis by associated gene or transcription factor. Each point in the plot represents a different bio sample (i.e., a similar set of methylated sites to those inputted were found in liver cells expressing N in experiment X, vs. a similar set of methylated sites to those inputted were found in blood cells expressing N in experiment Y, with each of these observations being hypothetical y axis values shown as dots on the N x coordinate).
